# Screening for Fabry Disease-Related Mutations Among 829 Kidney Transplant Recipients

**DOI:** 10.3390/jcm13237069

**Published:** 2024-11-22

**Authors:** Marina Kljajic, Armin Atic, Ivan Pecin, Bojan Jelakovic, Nikolina Basic-Jukic

**Affiliations:** 1 Department of Internal Medicine, University Hospital Centre Zagreb, 10000 Zagreb, Croatia; 2 School of Medicine, University of Zagreb, 10000 Zagreb, Croatia

**Keywords:** chronic kidney disease, Fabry disease, genetics, kidney transplantation

## Abstract

**Background/Objectives**: Fabry disease (FD) is a genetic lysosomal storage disease caused by a pathogenic variant in GLA gene coding for a functional alpha-galactosidase A enzyme whose disfunction leads to globotriaosylceramide (Gb3) accumulation in cells, which results in multiple organ disorders. The aim of this study was to identify mutations associated with Fabry disease among 829 kidney transplant recipients and to investigate the correlation between the factors such as age, dialysis vintage, eGFR, proteinuria and corticosteroid dose and the deviations in alpha-galactosidase A and lyso-Gb3 levels. **Methods:** Dry blood spot samples were collected for genetic analysis. The GLA genetic variants were analysed by an amplicon-based next-generation sequencing approach in all female patients and in male patients with reduced alpha-galactosidase A levels. Alpha-galactosidase A and Lyso-Gb3 were not determined in female patients. Pearson’s correlation coefficient was used to assess the relationship between the above-mentioned factors with the activity of alpha-galactosidase A and Lyso-Gb3. **Results:** Genetic testing was performed in 476 patients, all female patients (334), 69 male patients with decreased level of alpha-galactosidase A activity, one male patient with alpha-galactosidase A levels above the quantification limit and 72 male patients with no interpretable results of alpha-galactosidase A activity due to preanalytical error. In 3 (0.4%) male patients, hemizygous mutations associated with Fabry disease were found, and those were c.427G&gt;A p.(Ala143Thr), c.1181T&gt;C p.(Leu394Pro), and c.352C&gt;T p.(Arg118Cys). The dose of corticosteroid therapy seemed to be positively correlated to alpha-galactosidase A activity and negatively to Lyso-Gb3 levels in blood. **Conclusions**: Genetic testing of individuals with chronic kidney disease and reporting of genetic variants associated with the Fabry phenotype are important to improve the overall knowledge of the disease. Further research is needed to define factors influencing levels of alpha-galactosidase A and Lyso-Gb3.

## 1. Introduction

Fabry disease (FD) is an X-linked genetic lysosomal storage disease caused by mutations in the GLA gene coding for a functional alpha-galactosidase A enzyme, which is important in the metabolism of sphingolipids [1]. Enzymatic disfunction leads to globotriaosylceramide (Gb3) accumulation in cells, which results in multiple organ disorders. The neural, cardiovascular, and renal systems as well as the skin and eyes are frequently affected. Due to the multisystemic clinical symptom diversity, Fabry disease still appears to be underdiagnosed despite the availability of genetic testing, with the average time from symptom onset to diagnosis being more than ten years [2,3]. The disease is usually diagnosed in childhood or adolescence, while end-stage renal disease (ESRD), which most commonly presents with proteinuria, develops in the 3rd to 4th decade of life. Gb3 deposits can be found in all glomerular cell types (podocytes, endothelial and mesangial cells) and lead to activation of the immune system, resulting in a release of proinflammatory cytokines, cell migration and, ultimately, to the destruction of podocytes, hypercellularity of the mesangium, remodelling of the vascular system and fibrosis [4]. Globotriaosylsphingosine (lyso-Gb3) is a deacylated form of Gb3 found in plasma. It is a specific biomarker used in the detection of FD [5]. High levels of it, combined with reduced alpha-galactosidase A function, suggest a strong likelihood of FD. Decreased alpha-galactosidase A activity, paired with normal lyso-Gb3 levels, suggests a reduced likelihood of the classical type of Fabry disease [5]. Due to the X-linked pattern of inheritance, the disease is more commonly diagnosed in males, in whom a test of alpha-galactosidase A activity may be the best way for a decisive diagnosis. In women, however, enzyme activity can be misleading as they may have normal residual activity, and a genetic test should be performed [1]. Timely diagnosis is crucial as enzyme replacement therapy is a treatment option. Data on kidney transplant patients with Fabry disease are scarce. The aim of this study was to identify patients with abnormal levels of alpha-galactosidase A, lyso-Gb3 and genetic variants associated with Fabry disease among 829 kidney transplant recipients (KTRs) as well as to explore potential risk factors associated with abnormal alpha-galactosidase A and lyso Gb3 levels.

## 2. Materials and Methods

**Figure 1:**
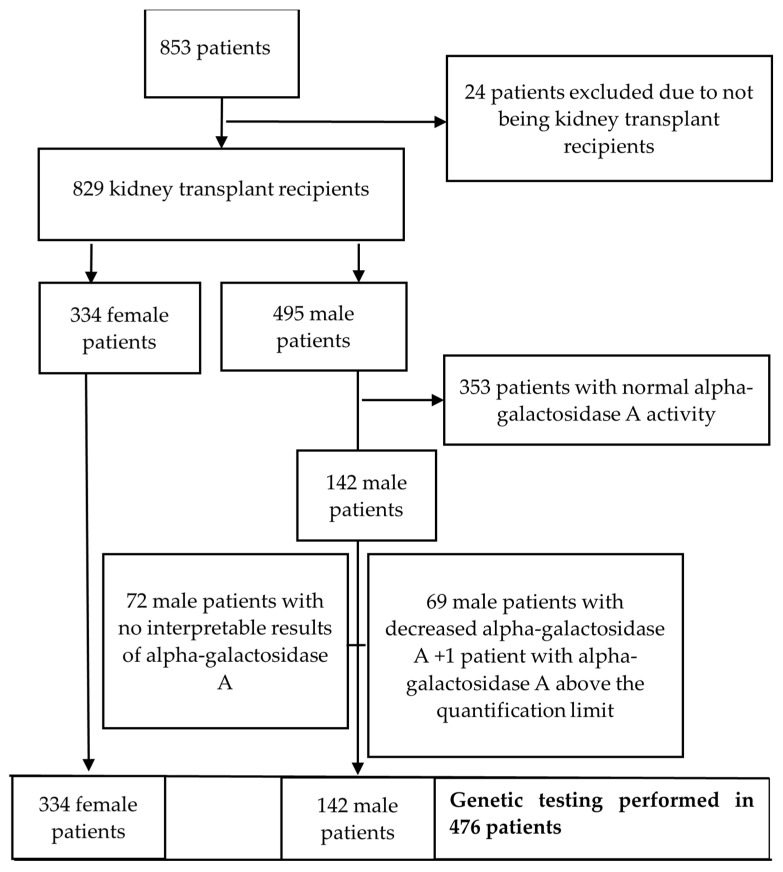
Flowchart illustrating the data reduction process.

This retrospective study included a total number of 853 patients whose dry blood spot samples were sampled for genetic analysis. Out of those 853, 24 of them were not KTRs and were therefore excluded from further analysis (Figure 1). Dry blood drop sampling was performed during a routine outpatient clinic appointment. Blood was transferred onto a piece of filter paper, left to dry at room temperature, and then kept at 22–24 °C until it was sent for analysis. Sample analysis was carried out in cooperation with the company Centogene (Rostock, Germany) and included the determination of alpha-galactosidase A activity and the level of lyso-Gb3 and genetic testing for mutations in the GLA gene. Activity of alpha-galactosidase A was determined by fluorimetry (normal range ≥ 15.3 µmol/L/h), while lyso-Gb3 was determined by liquid chromatography mass spectrometry (normal range ≤ 1.8 ng/mL). The GLA genetic variants were analysed by an amplicon-based next-generation sequencing approach in all female patients and in male patients with reduced alpha-galactosidase A levels. Alpha-galactosidase A and lyso-Gb3 were not determined in female patients. Patient characteristics (demographics, comorbidities, primary kidney disease, biopsy of native kidneys, dialysis vintage, type of dialysis, type of transplantation, maintenance immunosuppression regimens) were obtained from the hospital database. Proteinuria and estimated glomerular filtration rate (eGFR) values were measured in each patient at the time of blood sample taking for genetic analysis. For the missing data, complete-case analyses were used; i.e., subjects with missing data were excluded from the individual variable analysis. No input of missing data was performed, and this resulted in a different number of patients in the analysis of different variables. The primary objective of this study was to identify patients with mutations associated with Fabry disease and to investigate the correlation between the factors such as age, dialysis vintage, eGFR, proteinuria and corticosteroid dose and the deviations in alpha-galactosidase A and lyso-Gb3 levels. Pearson’s correlation coefficient was used to assess the relationship between the above-mentioned factors with the activity of alpha-galactosidase A and lyso-Gb3. A *p*-value of 0.05 or less was considered to be statistically significant.

## 3. Results

More males (59.7%) participated in the study than females (40.3%). The median value of the patients’ age was 59 years. In 38.3% of patients, biopsy of the native kidneys was performed prior to transplantation. Most first-time kidney transplantations were from deceased donors (92.3%), followed by living related (7.4%) and living unrelated donors (0.3%). Fifty-nine patients underwent a second kidney transplantation procedure, while a third transplantation was performed in only two patients. Renal function was replaced by haemodialysis in 69.2% of patients prior to transplantation. The majority of maintenance immunosuppressive regimens from the calcineurin inhibitor group included tacrolimus (73.5%), while the most frequently prescribed drug from the antimetabolite group of drugs was mycophenolate (94%). Corticosteroid dose varied from 2.5 (minimum) to 20 (maximum) mg.

Due to preanalytical failure, there were no reports on alpha-galactosidase A activity available in 72 male patients (Figure 1). Instead, lyso-Gb3 level determination and genetic analysis were performed in those patients. Preanalytical error occurred due to various reasons (heat, moisture, insufficient drying or incorrect preparation of the filter card) which led to deactivation of the enzymes in the sample. Alpha-galactosidase A level was within the normal range in 353 (83.5%) male patients, while 69 patients (16.3%) had reduced enzyme activity. One patient had alpha-galactosidase A levels above the quantification limit. Lyso-Gb3 levels were within the normal range in 141 patients (99.3%), except for 1 patient having elevated levels (Table 1).

Genetic testing was performed in 476 patients, all female patients (334), 69 male patients with decreased level of alpha-galactosidase A activity, 1 patient with alpha-galactosidase A activity above the quantification limit and 72 male patients with no interpretable results of alpha-galactosidase A activity due to preanalytical error (Figure 1). In 3 (0.4%) male patients, hemizygous mutations associated with Fabry disease were found, and those were c.427G&gt;A p.(Ala143Thr), c.1181T&gt;C p.(Leu394Pro), and c.352C&gt;T p.(Arg118Cys). The clinical characteristics of those patients are described in Table 2.

No significant correlations in terms of age, dialysis vintage, eGFR and proteinuria values with respect to alpha-galactose A and lyso-Gb3 levels were found. However, there seems to be a positive but weak, statistically significant correlation between corticosteroid dose and alpha-galactosidase A level and a weak negative correlation between corticosteroid dose and lyso-Gb3 levels in blood (Table 3).

## 4. Discussion

Our study found mutations in GLA genes c.427G>A p.(Ala143Thr), c.1181T>C p.(Leu394Pro), and c.352C>T p.(Arg118Cys) connected to FD in three male KTRs. More than 1100 mutations of the GLA gene that are associated with Fabry disease have been described in the literature to date [6,7]. To avoid the subjectivity of variant classification by individual laboratories, the American College of Medical Genetics and Genomics and the Association for Molecular Pathology (ACMG) published guidelines according to which all genetic variants should be categorised as follows: pathogenic, likely pathogenic, uncertain significance, likely benign, and benign [8].

In one of our patients, a hemizygous variant of the GLA gene, c.1181T>C p.(Leu394Pro), which causes an amino acid change from Leucine to Proline at codon 394, was found. This variant has not been registered in the Genome Aggregation Database and has been reported as a variant of uncertain significance in the ClinVar database [9,10]. This mutation was described in a female patient from the Fabry-Stroke Italian Registry who presented with an ischemic stroke at the age of 41 in compound heterozygosity with mutation c.427G>A, p.(Ala143Thr) [11,12]. Furthermore, one of the abstracts from the fourth Meeting of the Middle East North Africa Region reported on the mentioned mutation in a child with unspecified gender and decreased levels of alpha-galactosidase A [13]. In a Czech national FD screening study of patients undergoing chronic haemodialysis, a family screening identified 30 carriers (15 men and 15 women) with the same mutation, 75% of whom had at least one predefined FD manifestation. The most common manifestations were related to the renal and cardiovascular systems [14]. Our patient suffered from meningitis in early childhood, which is thought to be the reason for him being deaf and mute. In 1974, at 12 years of age, he was diagnosed with slowly progressive glomerulonephritis. In 2006, he was diagnosed with FD and started on ERT. It is important to note that this is the only patient who was diagnosed with FD prior to kidney transplantation. He had presented to a dermatological clinic several times because of angiokeratomas on his skin. In 2021, he reported to the emergency department due to peripheral facial nerve paresis. According to the latest cardiac ultrasound findings, his left ventricle has hypertrophic walls (14 mm) with preserved EF (60%). Both atria were described to be dilated. Alpha-galactosidase A activity in his blood sample was above the quantification limit (relative quantification: 985.2 μmol/L/h). This strongly increased activity was most likely due to sampling shortly after administration of ERT.

Genetic variant c.427G>A p.(Ala143Thr), also found in one of our patients, is a missense mutation that causes an amino acid change from alanine to threonine at position 143. There is a contradictory interpretation of the classification in the ClinVar database for this variant. The variant was reported in nine cases as a variant of unknown significance, in one case as likely pathogenic and in one case as likely benign. Only reports with applied ACMG classification criteria were counted [8,10]. In the literature, the mutation was described by Nance SC et al. as the cause of the late-onset Fabry disease which presented with cramp fasciculation syndrome in a 34-year-old patient [15]. Furthermore, it was seen in infants deficient in alpha-galactosidase A activity [16]. Interestingly, the grandfather of one of the affected babies suffered kidney failure of unknown aetiology at the age of 52 and developed a stroke 2 years later [16]. Terryn W et al. conducted a screening for FD mutations in individuals with left ventricular hypertrophy [17]. Variant c.427G>A p.(Ala143Thr) was identified in an adult male and three adult females. While the male patient showed symptoms affecting his nervous system and kidneys (renal insufficiency with proteinuria), his kidney biopsy did not reveal any Lyso-Gb3 deposits, unlike a female patient who had deposits in her myocardium. In another report (possibly with the same individuals), Teryn et al. reported no Gb-3 deposits in a cardiac biopsy in one female and kidney biopsies in two males [18]. Smid BE et al. concluded that the p.A143T mutation is not pathogenic [19]. They describe it in a family in which the mother had an asymptomatic cerebellar stroke and asymmetric cardiac hypertrophy. However, a myocardial biopsy failed to identify lamellated bodies characteristic of FD. Two of her sons had asymmetric septal hypertrophy; however, additional genetic screening revealed that they were carriers of a pathogenic mutation in the myosin-binding protein C gene [19]. Furthermore, Lenders M. et al. analysed 25 patients with the mutation and concluded that the mutation is likely to be a neutral variant or a potential modifier rather than a pathogenic mutation [20]. Prior to genetic testing, our patient was diagnosed with IgA nephropathy based on two native kidney biopsies performed during a diagnostic work-up for his renal insufficiency, which presented in 2010 with 24 h proteinuria of up to 6.8 g. His renal disease was stable until 2023 when he presented with symptoms of ESRD in the emergency department. He was on corticosteroid therapy for nine years after IgAN diagnosis and was lost to follow-up until 2023. During his HD treatment, he was referred to a neurologist because of frequent headaches. Brain magnetic resonance imaging and Color Doppler of the carotid arteries showed no abnormalities. Despite not undergoing ERT, his Lyso-Gb3 level was in the normal range. His family was reluctant to participate in further genetic testing.

The ClinVar database lists mutation c.352C>T p.(Arg118Cys) as a variant of uncertain significance in six reports with applied ACMG classification criteria [10]. The mutation was described in a case report of a woman treated for multiple angiokeratomas [21], as well as in a study that examined the prevalence of FD among young adult patients with stroke in Portugal [22]. When talking about neurologic manifestations, Lanthier S. et al. carried out a study in a cohort of 397 Canadian patients aged between 18 and 55 with a history of ischaemic stroke or episodes of transient ischaemic attack and found the mutation in a 55-year-old hemizygous man with no other symptoms related to Fabry disease [23]. The mutation was reported in an 8-year-old patient receiving ERT who experienced acroparesthesias, intense pain in the hands and feet, diarrhoea, constipation, bitemporal headache, dyshidrosis, recurrent fever and difficulty exercising with no major organ involvement [24]. Gaspar P. et al. identified the mutation in four ESRD patients in a study that examined 911 patients undergoing haemodialysis in Spain [25]. In addition, the mutation was described in a female patient with cystic kidneys and onset of ESRD at the age of 16, with extrarenal manifestations such as unexplained seizures, facial weakness, abdominal symptoms and photophobia [26]. When discussing renal cysts, which are relatively common in nephrological patients, the correlation between parapelvic cysts and FD described in the literature should be mentioned [27]. Therefore, the finding of parapelvic cysts in diagnostic imaging should indicate the possible diagnosis of FD [28]. The discussed mutation was also found in a 49-year-old male patient who underwent haemodialysis treatment for his end-stage renal failure. Two male grandchildren of the patient had characteristic signs of FD on a pathohistological report of renal biopsy despite having no evident signs of functional renal damage [29]. To prevent renal damage progression, they were started on ERT. According to Ferreira S. et al., the mutation modifies the multifactorial risk of cerebrovascular illness rather than having a Mendelian relationship with the clinical symptoms of FD [30]. However, the mutation has been linked to late-onset FD and hypertrophic cardiomyopathy [31,32,33]. Talbot A et al. found three family members (two sisters and a father) carrying the mutation through screening for familial cardiomyopathy [34]. All three of them displayed symptoms of reduced sweating, temperature analgesia, and low levels of alpha-galactosidase A. Additionally, in one of the sisters, occasional small and single lamellar bodies were found on cardiac biopsy [34]. Our patient underwent bilateral nephrectomy because of a bilateral clear cell carcinoma in 2011. During his pre-transplantation work-up in 2020, left ventricular concentric hypertrophy with normal contractility was noted on cardiac ultrasound, together with mild mitral and tricuspid insufficiencies. In 2018, he was diagnosed with suspected Gilbert syndrome due to a transient bilirubinaemia during an intentional weight loss episode. He has struggled with obesity since childhood. At the time of transplantation in 2020, his BMI was 37. When positive result of the genetic test came back, a brain MRI was performed and revealed no abnormalities. Furthermore, upon ophthalmological examination, no abnormalities connected to FD were found. In 2024, he developed bilateral pulmonary emboli and was treated as an outpatient. It is known for patients with FD to have an increased risk of thromboembolic events. According to Kang JJ. et al., alpha-galactosidase A deficiency may promote VWF secretion through decreased nitric oxide bioavailability and elevated reactive oxygen species, which increases the risk of thrombosis [35,36]. Our patient is not undergoing ERT and has stable graft function. His family members refused to participate in the screening for FD mutations. His father also underwent a nephrectomy due to renal carcinoma, while his paternal grandmother was treated with HD due to kidney atrophy.

Due to the complexity of the disease and the multiorgan involvement, an interdisciplinary approach is often required to establish the diagnosis [5]. It is important to consider this rare diagnosis and implement routine screenings among nephrological patients with unexplained proteinuria and glomerulopathy or early-onset kidney disease without typical risk factors [37]. Treatment nowadays focuses on prolonging the time to end-organ damage, reducing current symptoms and improving quality of life. Possible treatment options include chaperone and ER therapy, as well as symptomatic treatment that has no effect on the pathophysiology of the disease [38]. All three of our patients with Fabry disease-associated mutations had normal levels of lyso-Gb3 in their blood. In one of the patients, this could be due to the fact that he was undergoing active ERT treatment at the time of blood sampling, as ERT has been shown to reduce the accumulation of Gb3 in tissues [39,40]. Due to a broad genotypic and phenotypic diversity, management of the disease requires a multisystemic assessment and a personalised therapeutic approach for each individual patient [38]. It is recommended that patients with mutations associated with FD undergo regular clinical assessments [5]. Phenotypic expression should be monitored, and the therapeutic approach should be tailored according to the disease burden. Timely initiation of treatment is of crucial importance because it stabilises kidney function and delays irreversible end-organ damage [41,42]. Furthermore, reducing modifiable cardiovascular risk factors such as arterial hypertension, dyslipidaemia, obesity and smoking is recommended [5]. Genetic testing should be offered to family members of the affected individual, since it may lead to the identification of affected individuals who may benefit from therapeutic intervention. Counselling of family members before and after genetic testing is important, as the diagnosis of FD often causes anxious–depressive symptoms among the affected individuals and their family [43]. In the context of kidney transplantation, genetic testing of family members is essential for planning a live donor transplant from a family member [44].

Kidney transplantation is considered safe and beneficial for patients with Fabry disease [45]. According to a recently published meta-analysis, which included 55 studies and a total of 84,062 individuals, the prevalence of Fabry disease patients among renal transplant recipients seems to be 0.28%, which is less than in our study (0.4%) [46]. However, our study included a smaller number of KTRs. In our study population, no genetic variants were noted in female patients. In a recently published screening study of 301 KTRs, Erdogmus S. et al. found 1 male patient with a pathogenic mutation (0.3%) and 2 female patients with a GLA mutation of unknown significance (0.6%) [47]. To our knowledge, this is the first study to examine the influence of factors such as age, dialysis vintage, eGFR and proteinuria on alpha-galactose A and Lyso-Gb3 levels in KTRs. According to the results of our study, the dose of corticosteroid therapy is positively correlated to alpha-galactosidase A activity and negatively to Lyso-Gb3 levels in blood, which cannot be explained based on the available literature. Interestingly, Hasbal N.B. et al. reported lower alpha-galactosidase A levels in patients treated for focal segmental glomerulosclerosis (FSGS) than in patients undergoing HD [48]. It cannot be determined whether this is related to corticosteroid therapy, which may be used in the treatment of FSGS, as no detailed information on patient treatment was provided. Although there are no data in the literature on this, one possible hypothesis linking corticosteroid therapy to changes in α-Gal A levels may be due to the possible effects of corticosteroids on enzyme activity, the lysosomal storage system and glycosphingolipid metabolism. However, further studies are needed to establish a link between corticosteroids and alpha-galactosidase A levels, ideally in a population of patients treated with corticosteroids for other diseases.

## 5. Conclusions

Fabry disease is rare among kidney transplant recipients. Further research is needed in order to define factors influencing the levels of alpha-galactosidase A and lyso-Gb3 in blood. According to the current ACGM classification, the results of this study do not prove the presence of Fabry disease in these individuals. However, the reporting of genetic variants associated with the Fabry phenotype is important to improve the overall knowledge of this rare disease. Genetic counselling is to be offered to all patients and their families with evidence of genetic variants in the GLA gene.

## Figures and Tables

**Table 1:** Patients’ characteristics.

Sex [n (%)]		
Male	495 (59.7)	
Female	334 (40.3)	
Age (years) [Median (IQR)]	59 (45–68)	min 19 max 87
PHD of native kidney disease (n = 798) [n (%)]		
No	492 (61.7)	
Yes	306 (38.3)	
Donor type—1st Tx (n = 822) [n (%)]		
Deceased	759 (92.3)	
Living related	61 (7.4)	
Living unrelated	2 (0.3)	
Donor type—2nd Tx (n = 59) [n (%)]		
Deceased	55/59 (93.2)	
Living related	4/59 (6.8)	
Donor type—3rd Tx (n = 2)		
Deceased	1/2	
Living unrelated	1/2	
Type of dialysis [n (%)] (n = 829)		
Pre-emptive	28 (3.4)	
HD	574 (69.2)	
PD	132 (16.0)	
HD + PD	95 (11.4)	
Dialysis vintage (months) [Median (IQR)]	33 (16–56)	min 0 max 300
eGFR [Median (IQR)]	51 (35–67)	min < 15 max 138
Proteinuria/24 h [Median (IQR)]	213 (113–428.3)	min 25 max 10,072
CNI [n (%)] (n = 724)		
Tacrolimus	532 (73.5)	
Cyclosporine	192 (26.5)	
mTORi [n (%)] (n = 173)		
Everolimus	166 (96)	
Sirolimus	7 (4)	
Antimetabolites [n (%)] (n = 703)		
Mycophenolate	661 (94)	
Azathioprine	42 (6)	
Corticosteroid dose (n = 829) [Median (IQR)]	5 (5–5)	min 2.5 max 20
Alpha-gal A activity (µmol/L/h) (n = 422)	19 (16.1–23.5)	min 6.8 max 101.0
Alpha-gal A [n (%)] (n = 423)		
Reference value (≥15.3 µmol/L/h)	353 (83.5)	
<15.3 µmol/L/h	69 (16.3)	
Lyso Gb3 level (ng/mL) (n = 142)	1.25 (1.10–1.43)	min 0.60 max 1.90
Lyso Gb3 [n (%)] (n = 142)		
Reference value (≤1.8 ng/mL)	141 (99.3)	
>1.8 ng/mL	1 (0.7)	
Fabry disease-related mutation [n (%)]	3 (0.4)	
GLA mutation testing [n (%)] (n = 476)		
no clinically relevant variant	473 (99.4)	
GLA, c.427G>A p.(Ala143Thr)	1 (0.2)	
GLA, c.1181T>C p.(Leu394Pro)	1 (0.2)	
GLA, c.352C>T p.(Arg118Cys)	1 (0.2)	

PHD—pathohistological diagnosis; Tx—transplantation; HD—haemodialysis; PD—peritoneal dialysis; eGFR—estimated glomerular filtration rate; CNI—calcineurin inhibitor; mTORi—mammalian target of rapamycin inhibitors; alpha-gal A—alpha-galactosidase A; lyso-Gb3—globotriaosylsphingosine; min—minimal value; max—maximal value.

**Table 2:** Characteristics of patients with mutations connected to Fabry disease.

Patient	Sex	Age	Alpha-Galactosidase A (µmol/L/h)	Lyso-Gb3 (ng/mL)	GLA Mutation	Kidney Biopsy	Diagnosis Prior to Testing	Other Non-Renal Manifestations	Follow Up	Centogene’s Classification of Variant Based on ACMG Guidelines
1	M	61	>100	0.9	c.1181T>C p.(Leu394Pro)	No	Chronic GN	LVH, paraesthesia, angiokeratomas, deafness	Preserved graft function, on regular ERT.	Likely pathogenic
2	M	32	6.8	1.5	c.427G>A p.(Ala143Thr)	Yes	IgAN	Headaches	Preserved graft function, not on ERT.	VUS
3	M	45	7.8	1.4	c.352C>T p.(Arg118Cys)	Yes	Renal carcinoma	PE in 2024, LVH, muscle/joint pain	Preserved graft function, not on ERT.	VUS

M—male; IgAN—IgA nephropathy; LVH—left ventricular hypertrophy; ERT—enzyme replacement therapy; PE—pulmonary embolism; GN—glomerulonephritis; VUS—variant of uncertain significance; ACMG—American College of Medical Genetics and Genomics and the Association for Molecular Pathology.

**Table 3:** Correlation between age, dialysis vintage, eGFR, proteinuria and corticosteroid dose with alpha-gal A and Lyso Gb3 levels.

	Pearson’s Correlation Coefficient R (*p* Value)		95% Confidence Interval (CI)	
	Alpha-Galactosidase A Activity	Lyso Gb3 Level	Lower	Upper
Age	−0.094 (0.05)	0.154 (0.08)	−1488	2550
Dialysis vintage (months)	0.002 (0.97)	0.002 (0.98)	−10,873	1166
eGFR	−0.092 (0.06)	0.088 (0.32)	−4657	1620
Proteinuria	0.054 (0.26)	−0.055 (0.54)	40,956	282,714
Corticosteroid dose	0.158 (0.001)	−0.189 (0.03)	0.1075	0.8724

## Data Availability

The raw data supporting the conclusions of this article will be made available by the authors upon request.
